# Epigenomic landscape exhibits interferon signaling suppression in the patient of myocarditis after BNT162b2 vaccination

**DOI:** 10.1038/s41598-023-36070-y

**Published:** 2023-06-01

**Authors:** Hyeonhui Kim, Hyo-Suk Ahn, Nahee Hwang, Yune Huh, Seonghyeon Bu, Kyung Jin Seo, Se Hwan Kwon, Hae-Kyung Lee, Jae-woo Kim, Bo Kyung Yoon, Sungsoon Fang

**Affiliations:** 1 Graduate School of Medical Science, Brain Korea 21 Project, Yonsei University College of Medicine, Seoul, 03722 Korea; 2 Severance Biomedical Science Institute, Gangnam Severance Hospital, Yonsei University College of Medicine, Seoul, 03722 Korea; 3 Division of Cardiology, Department of Internal Medicine, The Catholic University of Korea, Uijeongbu St. Mary’s Hospital, Seoul, 06591 Korea; 4 Catholic Research Institute for Intractable Cardiovascular Disease (CRID), College of Medicine, The Catholic University of Korea, Seoul, 06591 Korea; 5 Department of Biochemistry and Molecular Biology, Yonsei University College of Medicine, Seoul, 03722 Korea; 6 Department of Medicine, Yonsei University College of Medicine, Seoul, South Korea; 7 Department of Hospital Pathology, College of Medicine, The Catholic University of Korea, Uijeongbu St. Mary’s Hospital, Seoul, South Korea; 8 Department of Radiology, Kyung Hee University Medical Center, Seoul, South Korea

**Keywords:** Next-generation sequencing, Epigenetics in immune cells, RNA vaccines

## Abstract

After the outbreak of the severe acute respiratory syndrome coronavirus 2 (SARS-CoV-2) pandemic, a novel mRNA vaccine (BNT162b2) was developed at an unprecedented speed. Although most countries have achieved widespread immunity from vaccines and infections, yet people, even who have recovered from SARS-CoV-2 infection, are recommended to receive vaccination due to their effectiveness in lowering the risk of recurrent infection. However, the BNT162b2 vaccine has been reported to increase the risk of myocarditis. To our knowledge, for the first time in this study, we tracked changes in the chromatin dynamics of peripheral blood mononuclear cells (PBMCs) in the patient who underwent myocarditis after BNT162b2 vaccination. A longitudinal study of chromatin accessibility using concurrent analysis of single-cell assays for transposase-accessible chromatin with sequencing and single-cell RNA sequencing showed downregulation of interferon signaling and upregulated RUNX2/3 activity in PBMCs. Considering BNT162b2 vaccination increases the level of interferon-α/γ in serum, our data highlight the immune responses different from the conventional responses to the vaccination, which is possibly the key to understanding the side effects of BNT162b2 vaccination.

## Introduction

Severe acute respiratory syndrome coronavirus 2 (SARS-CoV-2) has infected more than 600 million patients, resulting in more than 6 million deaths worldwide. The cumulative number of SARS-CoV-2 vaccines administered has surged to almost 130 million^1^. One of the most administered vaccines is the Pfizer-BioNTech BNT162b2 mRNA. Despite its effectiveness in protection against SARS-CoV-2 infection, the BNT162b2 vaccine has recently been reported to be associated with an increased risk of myocarditis. Its pathogenesis remains unknown^2,3^.

Recent studies on the immune response to SARS-CoV-2 infection have identified important transcriptomic signatures^4^, which include changes in genes involved in interferon (IFN) signal transduction and natural killer (NK) cell maturation^5^. Along with clinical findings that indicate type I IFN deficiency in the blood of severe SARS-CoV-2 patients^6,7^, recombinant type I IFN, although controversial, has brought attention as a potential therapeutic agent for SARS-CoV-2 infection^8^. Compared with other respiratory viruses, SARS-CoV-2 infection leads to weak IFN (type I and III) responses while inducing robust expression of interleukin-6 and chemokines^9^. Low innate antiviral defenses resulting from low levels of IFN have been considered a driving feature of SARS-CoV-2 infection^9^.

However, there are relatively few studies focusing on the characterization of the immune landscape of patients showing side effects after BNT162b2 administration despite the surging number of shots. To our knowledge, currently, there are no studies identifying the epigenomic landscape of immune cells of BNT162b2-induced myocarditis patients in single-cell resolution, which may provide deep insight regarding the pathogenesis of vaccine-induced myocarditis. Here, we applied a single-cell sequencing assay for transposase-accessible chromatin (scATAC-seq) with single-cell RNA sequencing (scRNA-seq) in a patient with BNT162b2-induced myocarditis at the acute inflammatory stage and after remission. A comprehensive understanding of changes in the immune landscape accompanied by BNT162b2-induced myocarditis showed global upregulation of RUNX and downregulation of IFN.

## Results

### Overview of longitudinal analysis in single-cell resolution from peripheral blood mononuclear cell (PBMCs) of a patient with myocarditis after BNT162B2 vaccination

**Figure 1:**
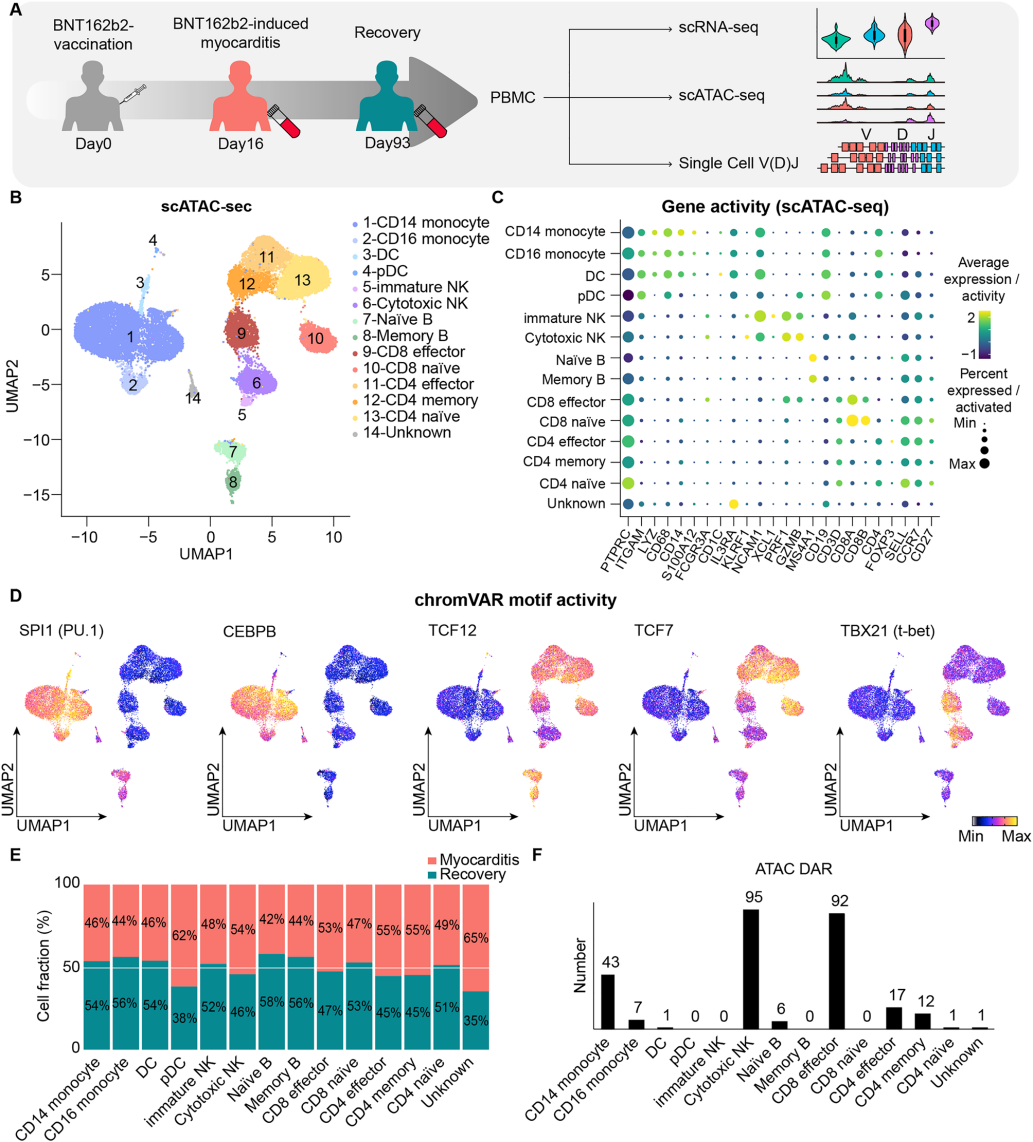
Overview of Integrated analysis of transcriptomic and epigenomic signatures of peripheral immune cells in the patient of myocarditis after BNT162b2 vaccination. (**A**) Overview of the experiment. Single-cell sequencing assay for transposase-accessible chromatin (scATAC-seq), scRNA-seq, and single-cell VDJ analysis were performed using peripheral blood mononuclear cells peripheral blood mononuclear cells (PBMCs) from patients with acute myocarditis (day 16) after BNT162b2 vaccination and recovery conditions (day 93). (**B**) Uniform manifold approximation and projection (UMAP) plot representing cluster annotation of single-cell sequencing assay for transposase-accessible chromatin (scATAC-seq). (**C**) Dot plot showing canonical immune cell marker gene activity of the single-cell sequencing assay for transposase-accessible chromatin (scATAC-seq) datasets. Gene activity was calculated as chromatin accessibility of the promoter and gene body. The diameter corresponds to the population percentage of cells calculated gene activity in the subtype. The average gene activity level of the cell subtype appears as a color gradation. (**D**) Single-cell sequencing assay for transposase-accessible chromatin (scATAC-seq) uniform manifold approximation and projection (UMAP) plot representing chromVAR motif activity of transcription factors involved in immune cell activation and differentiation. The color gradient represents the chromVAR TF motif bias-corrected deviations. (**E**) The fraction of cell clusters was calculated using 10x-based single-cell sequencing assay for transposase-accessible chromatin (scATAC-seq). (**F**) The number of differentially accessible regions (DARs) of myocarditis versus recovery in each immune cell subtype was counted (logFC > 0.25, adjusted *P* value < 0.05, minimum percentage of expressing cells > 10%).

Previously, we had reported the peripheral immune landscape of a 59-year-old male patient who had developed severe myocarditis after BNT162b2 vaccination^10^. Blood was collected at two time points: immediately after the onset of myocarditis and after the patient completely recovered from myocarditis. The immune response of patients with myocarditis after BNT162b2 vaccination was analyzed in more detail by comparing vaccinated individuals without side effects, Coronavirus disease 2019 (COVID-19) patients and Kawasaki disease patients at the single-cell level^11^. To further analyze the dynamic changes in the epigenomic landscape associated with myocarditis resulting from BNT162b2 vaccination, we performed a parallel single-cell sequencing assay for transposase-accessible chromatin (scATAC-seq), in addition to transcriptomic analysis, on two PBMC samples collected at different time points (Fig. 1A). We obtained scATAC-seq datasets from 20,519 PBMCs collected at two time points after quality control.

We evaluated batch correction through dimensionality reduction uniform manifold approximation and projection (UMAP) and performed graph-based clustering on scRNA-seq and scATAC-seq, which identified 25 and 16 clusters, respectively (Fig. S1A-C). Each scRNA-seq cluster was annotated based on the transcriptional profiles of canonical immune cell markers (Fig. S1D, and S1E). The scATAC-seq clusters were initially identified using the label transfer function of Signac *TransferData* and further identified by gene activity and computed by counting the number of sequenced fragments overlapping with the gene body and a 2 kb upstream region from transcription start sites (TSS) for each gene (Figs. 1B,C, S1F, and S1G). Next, we verified cell annotations using chromVAR motif activities of the regions important in lineage-specific differentiation and activation. The motifs used for annotation verification were as follows: SPI1 (PU.1) for myeloid and B cells^12^, CEBPB for monocytes^13^, TCF12 for B cells^14^, TCF7 for T cells^15^, and TBX21 (t-bet) for CD4 T cells, CD8 T cells, NK cells, and B cells^16^ (Fig. 1D). Co-embedding scRNA-seq and scATAC-seq data into a single UMAP visualization validated cluster annotations with a high overlap rate (Fig. S2A–E).

Next, we analyzed the distribution of immune cell populations at different time points. The fraction of each cluster presented minimal change between myocarditis and recovery states, except for plasmacytoid dendritic cell (pDC), which is the cluster with the smallest cell number (Figs. 1E, and S3A). Thus, the cell fractions of most immune cells were similar between the two time points, indicating that immune cell composition is not a key contributing factor for the development of myocarditis. We computed the differentially accessible region (DAR) of each cluster (Fig. 1F). In contrast to the differentially expressed genes (DEGs) in scRNA-seq, chromatin accessibility was most dynamically changed in cytotoxic NK and CD8 effector T cells in scATAC-seq (Fig. S3B). The location of the DAR was annotated with clusterProfiler (Fig. S3C and S3D). We performed single-cell TCR sequencing (scTCR-seq) analysis to investigate T-cell immunity. However, the most abundant complementarity-determining region 3 (CDR3) sequence showed minimal changes (Fig. S3E). In contrast, we observed increased gene activities of immune activation marker genes^17^ in the CD8 effector and cytotoxic NK cluster at myocarditis state (Fig. S4A–C). Furthermore, the up-regulation of motif activity and gene activity in fatty acid metabolism related genes at acute myocarditis validated the scATAC-seq data by demonstrating consistency with the results of the scRNA-seq analysis^11^ (Fig. S5A–D).

### Upregulation of RUNX transcriptional activity at the acute myocarditis stage

**Figure 2:**
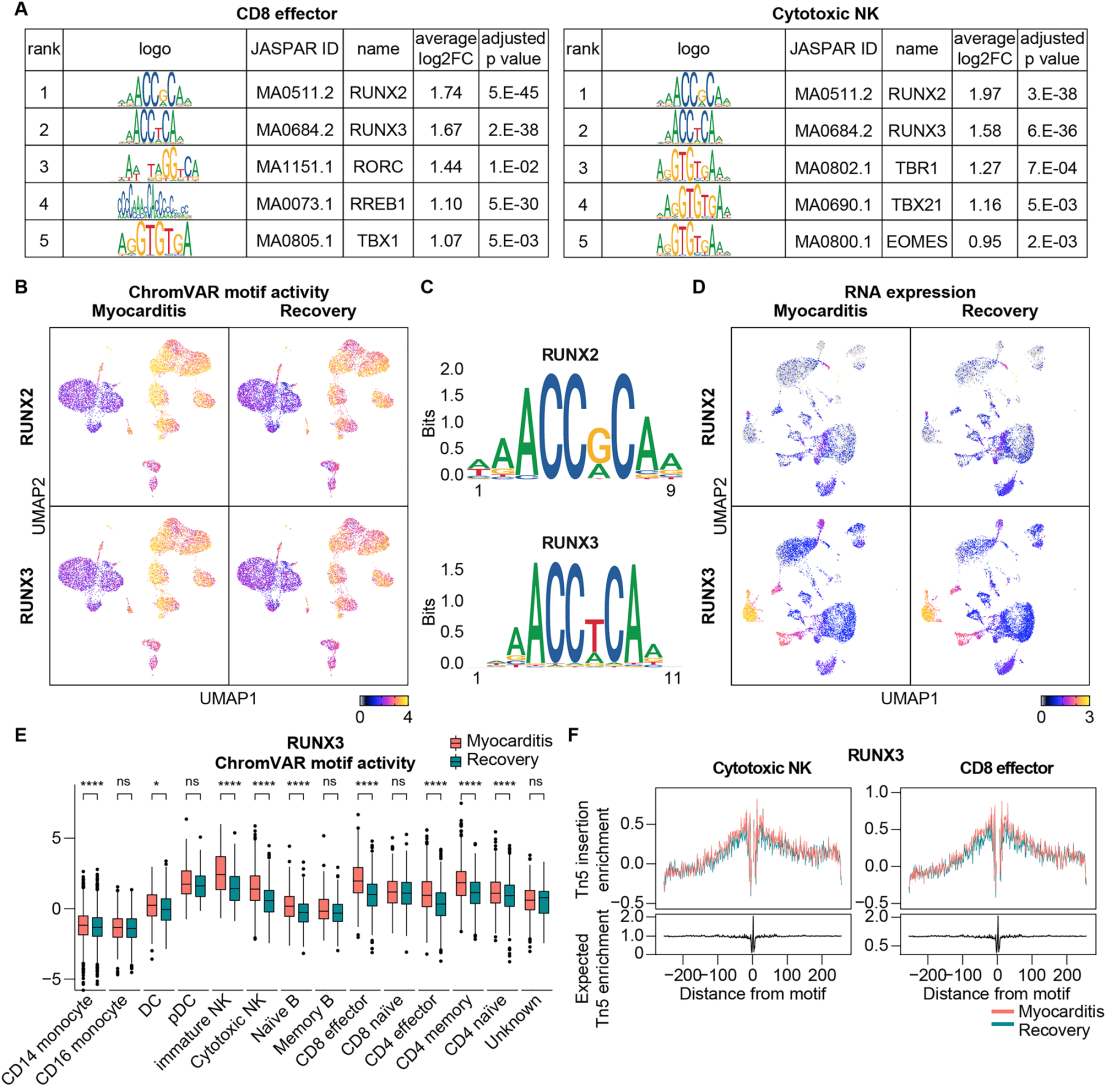
RUNX transcriptional activity is upregulated in the diverse immune cells at the acute myocarditis stage. (**A**) The table shows the top five enriched motifs in CD8 effector and cytotoxic NK cells. We performed chromVAR motif analysis using differentially accessible regions (DARs) of each cluster. (**B**) Uniform manifold approximation and projection (UMAP) plot of single-cell sequencing assay for transposase-accessible chromatin (scATAC-seq) with chromVAR motif activity of RUNX2 and RUNX3. The color gradient represents the chromVAR TF motif bias-corrected deviations. (**C**) Plot of the position weight matrices for the motifs of RUNX2 and RUNX3. (**D**) Uniform manifold approximation and projection (UMAP) plot of scRNA-seq with the RNA expression of RUNX2 and RUNX3. Color gradient represents log normalized gene expression. (**E**) The box plot shows RUNX3 chromVAR motif activity of the immune cell subtype. (**F**) TF footprints of RUNX3 in the cytotoxic NK and CD8 effector subtypes. The Tn5 insertion bias track is also shown.

Changes in chromatin openness can modulate the availability of binding sites for TFs and control gene expression^18^. To assess the binding affinity of TFs, we used chromVAR, which calculates the bias-corrected TF accessibility deviation for each motif across genome-wide signals^19^. According to chromVAR analysis, RUNX2 and RUNX3 motifs are globally upregulated across most cell types during acute myocarditis in terms of motif activity (Fig. 2A and B). The RUNX family is a key regulator of development and differentiation, especially of blood cells^20^. Furthermore, recent evidence suggests the importance of RUNX in the immune response against pathogens, including viruses, such as the Epstein-Barr virus and Influenza A^21,22^.

The RUNX family has a highly conserved DNA-binding domain and shares a consensus-binding motif sequence^23^ (Fig. 2C). To find the major RUNX gene important in myocarditis pathogenesis, we compared the expression levels of RUNX2 and RUNX3 (Fig. 2D). Although RUNX2 and RUNX3 showed upregulation of their motif activities in myocarditis, their mRNA expression levels indicated minimal changes between the two time points, highlighting the importance of ATAC-seq for understanding the gene-modulatory network. Since RUNX3 exhibits higher expression levels across all cell subpopulations than RUNX2, we propose RUNX3 as the primary TF upregulated during acute myocarditis. Notably, the accessibility of the chromatin regions overlapping with the ENCODE ChIP-seq peak of RUNX3 increased in the myocarditis state (Fig. S6).

RUNX3 is involved in the interleukin-15-dependent activation of NK cells^24^ and the proliferation and cytotoxicity of CD8 T cells^25^. RUNX3 was also highly enriched in NK and T cells during myocarditis in terms of motif activity (Fig. 2E), and increased accessibility of RUNX3 was confirmed across the whole genome (Fig. 2F). Thus, the TF RUNX3 showed the highest increase in activity during myocarditis.

### Suppression of type 1 IFN signaling at the acute myocarditis stage

**Figure 3:**
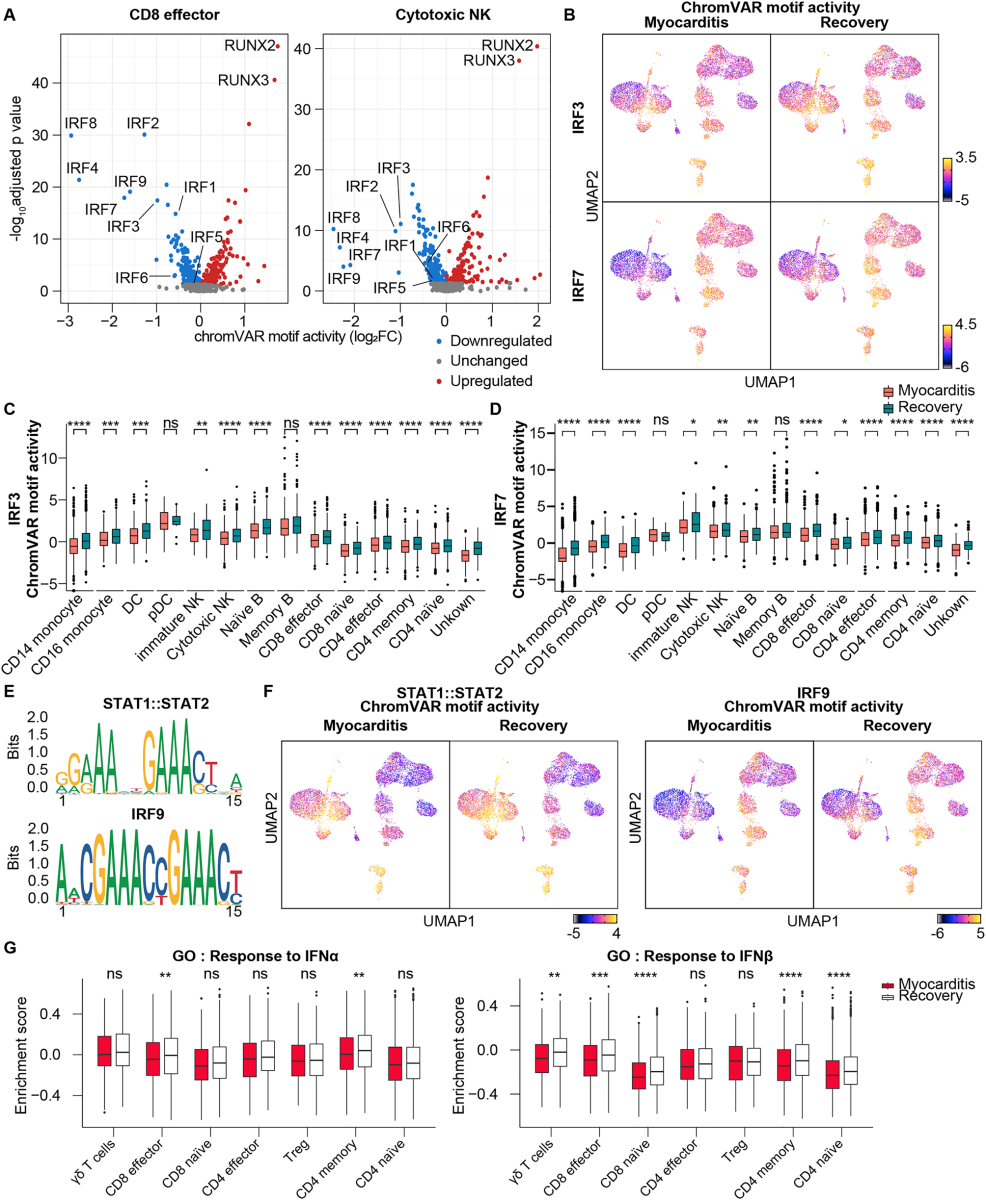
Type 1 interferon (IFN) signaling is downregulated at the acute myocarditis stage. (**A**) Volcano plot shows the differential motif activities using the mean motif activity. The x-axis represents the difference in mean motif accessibility calculated using the chromVAR TF bias-corrected deviation in CD8 effector and cytotoxic NK cell clusters. (**B**) Uniform manifold approximation and projection (UMAP) plot of single-cell sequencing assay for transposase-accessible chromatin (scATAC-seq) with chromVAR motif activity of IRF3 and IRF7. The color gradient represents the chromVAR TF motif bias-corrected deviations. (**C**, **D**) Box plot showing the IRF3 and IRF7 chromVAR motif activity of the immune cell subtype. (**E**) Position weight matrices for the motifs of the STAT1 and STAT2 heterodimers (STAT1::STAT2) and IRF9. (**F**) Uniform manifold approximation and projection (UMAP) plot of single-cell sequencing assay for transposase-accessible chromatin (scATAC-seq) overlaid chromVAR motif activity of STAT1::STAT2 and IRF9. The color gradient represents the chromVAR TF motif bias-corrected deviations. (**G**) Box plot representing the GSVA score performed to calculate enrichment with GO pathway “GO:0035455_RESPONSE_TO_INTERFERON_ALPHA” and “GO:0035456_RESPONSE_TO_INTERFERON_BETA” from MSigDB v7.4.

Next, we investigated TFs whose activities were downregulated at the time of acute myocarditis in the cell subpopulations with the highest number of DEGs. In NK cells and CD8 effector cells, IFN regulatory factors (IRFs) showed the highest degree of downregulation in the myocarditis state (Fig. 3A). IRF family consists of nine members (IRF1–IRF9) and plays a crucial role in IFN production and response against viral infection and inflammation^26^. IFN, a pleiotropic cytokine that regulates the immune response, has two major families: type I IFN (IFNα and IFNβ) and type II IFN (IFNγ). During viral infection, viral DNA and RNA activate IRF3/IRF7, which activates IFNA/IFNB gene transcription^27,28^. Secreted IFNα or IFNβ binds to the IFN-α/β receptor on the surface of almost all cell types, leading to the formation of the STAT1/STAT2/IRF9 complex, known as the ISGF3 complex. This complex initiates the transcription of IFN-stimulated genes to stimulate an immune response to eliminate the viral infection.

Previous studies have reported a decrease in the level of type I IFNs in the blood and the amount of type 1 IFN production from blood immune cells in patients with severe symptoms of COVID-19^6,29^. In our datasets, although the gene activity and RNA expression level of IRF3 showed minimal changes (Figs. S7A, and B), the motif activity of IRF3 and IRF7 was decreased at myocarditis state across all cell subpopulations except for pDCs and memory B cells (Figs. 3B–D, and S7C). When we compared the openness of genomic regions predicted to be the sites of IRF3/7 binding, chromatin accessibility was reduced in myocarditis CD8 effector and cytotoxic NK cells (Fig. S8A, and B).

In addition to IFNα/β production, we investigated changes in motif activity in IFNα/β receivers. IFNα/β induced the transcription of IFNα/β response genes by the activation of the ISGF3 complex, which consists of a heterodimer of STAT1 and STAT2 (STAT1::STAT2) and IRF9 (Fig. 3E). Consistent with the downregulation of motif activities of IRF3 and IRF7, peripheral immune cells showed a reduced motif activity level of STAT1::STAT2 and IRF9 at the time of myocarditis (Fig. 3F). In addition, we performed Gene Set Variation Analysis (GSVA), which calculates gene set enrichment scores for a sample to validate changes in RNA expression profile in response to Type I IFN^30^. A decrease in IFNα/β-related gene expression was primarily observed in T cells. IFNβ had a more profound influence on the immune cell response in myocarditis (Fig. 3G).

### Suppression of type 2 IFN signaling at the acute myocarditis stage

The only member of the type II IFN family, IFNγ, is essential for the inflammatory response triggered by viral infections. Adaptive immune cells, including CD4 T helper type 1 cells, γδT cells, activated NK cells, and cytotoxic CD8 T cells, secrete IFNγ upon viral infection. Secreted IFNγ induces nuclear entry of the STAT1 homodimer into immune cells and initiates the transcription of primary response genes, such as IRF1.

**Figure 4:**
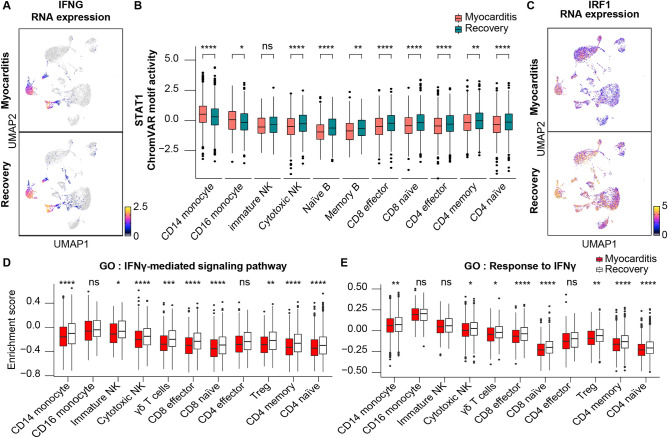
Type 2 interferon (IFN) signaling is downregulated at the acute myocarditis stage. (**A**) The uniform manifold approximation and projection (UMAP) plot of scRNA-seq overlaid the RNA expression of IFNG. Color gradient represents log normalized gene expression. (**B**) Box plot showing the STAT1 chromVAR motif activity of the immune cell subtype. (**C**) Uniform manifold approximation and projection (UMAP) plot of scRNA-seq overlaying the RNA expression of IRF1. Color gradient represents log normalized gene expression. (**D**, **E**) Box plot representing Gene Set Variation Analysis (GSVA) score performed to calculate enrichment with the GO pathway “GO:0060333_INTERFERON_GAMMA_MEDIATED_SIGNALING_PATHWAY” and “GO:0034341_ GOBP_RESPONSE_TO_INTERFERON_GAMMA” from MSigDB v7.4.

We first investigated the mRNA expression levels and distribution patterns of IFN-γ. Cytotoxic NK and CD8 T cells showed abundant expression and decreased expression levels at the time of myocarditis, respectively (Figs. 4A, S9A). In addition, there was a mild decrease in chromatin accessibility with a reduction in mRNA expression level at the region, which *Cicero* predicts as a *ci*s-regulatory DNA region for IFNG transcription (Fig. S9B).

Considering IFNγ signaling-recipient cells, decreased motif activity in STAT1 across multiple cell types in myocarditis suggests that the response to IFNγ secreted from cytotoxic NK and CD8 effector cells was downregulated (Fig. 4B, S9C, and D). IRF1, a target gene of the IFNγ signaling pathway, was also downregulated at the myocarditis state (Figs. 4C, and S9E). The GSVA score analyzed from mRNA expression levels advocates blunted response to IFNγ in CD14 monocyte, cytotoxic NK, CD8 T, Treg, CD4 memory, and CD4 naïve cells (Fig.4D and E). Therefore, the immune system of a patient with acute myocarditis after BNT162b2 vaccination exhibited impaired IFN signaling, although aggregated peaks in the regulatory element of pro-inflammatory cytokine were increased (Fig. S10). Considering that BNT162b2 vaccination induces the upregulation of serum IFN levels, the patient with the side effect of vaccination is likely to develop immune responses different from what has been known^31^.

## Discussion

Inferring molecular dynamics during disease progression is challenging, particularly when there are large time gaps between sampling points^32^ Analysis based only on the expression levels is not sufficient to map the major driving force, such as key TFs, in the longitudinal analysis of the patient. Investigation of chromatin accessibility is an effective tool for studying active regulatory DNA^33^.

**Figure 5:**
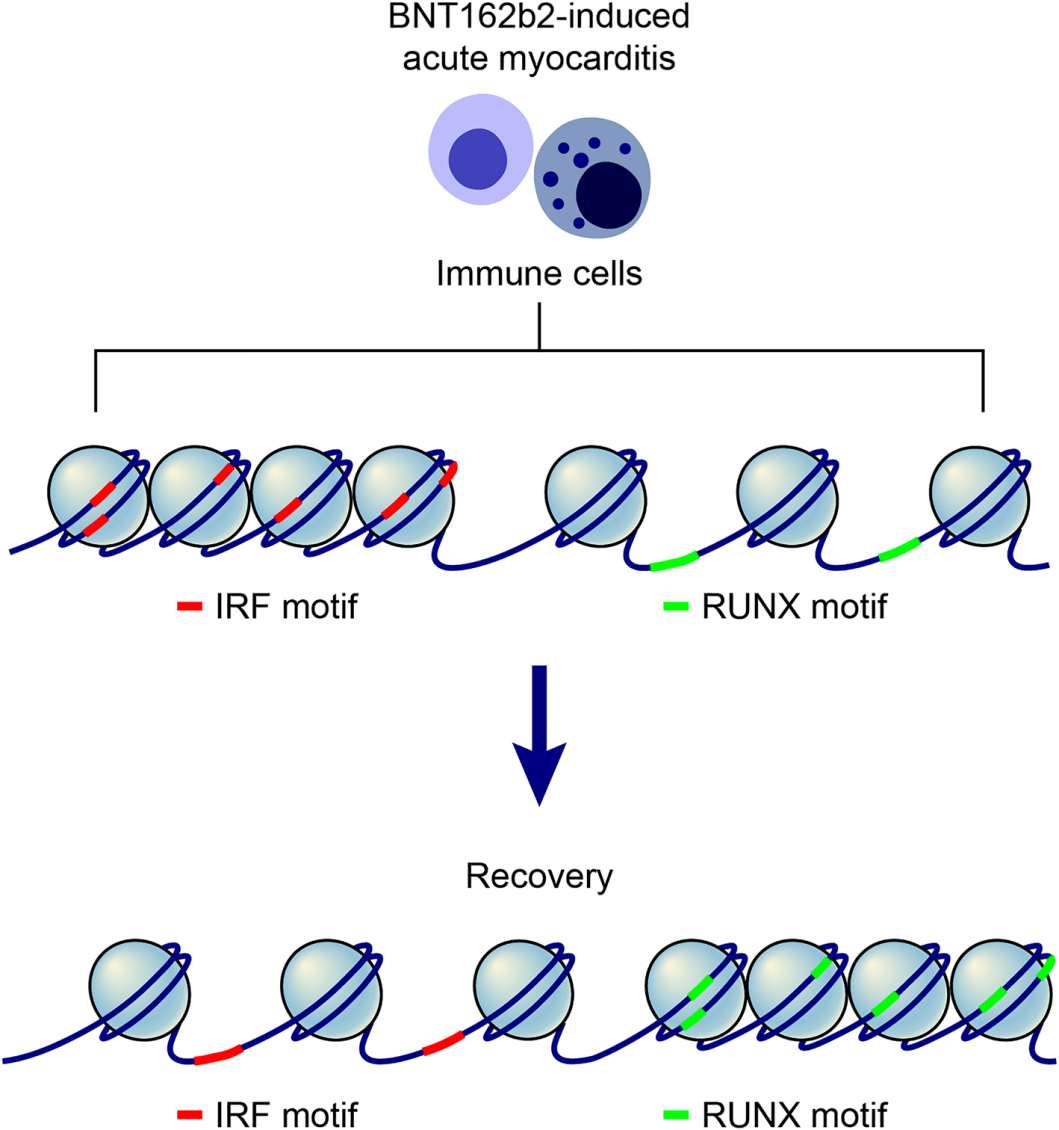
The proposed model of how chromatin structure differs at the acute BNT162b2-induced myocarditis stage.

Gene expression levels can typically be assessed by measuring the amount of mRNA transcripts and the binding efficiency of gene modulators to the promoter region. However, the role of regulatory elements in controlling target gene expression has recently been highlighted in various biological processes^33^. Thus, TF activities can be comprehensively estimated by changes in chromatin accessibility of the regions across promoters and cis-regulatory elements that affect the binding of TFs to motifs^19^. Here, we found that gene expression modulators were differentially regulated at the time of myocarditis (Fig. 5). In addition, the activity of genes that could not be identified in the sparse RNA matrix can be predicted through the successful analysis of chromatin accessibility in the gene body, promoter, and cis-elements^33,34^.

Integrated analysis of scRNA-seq and scATAC-seq from our results revealed a downregulated IFN signaling pathway and upregulated gene activities of IL-1, IL-6, IL-17, and IL-21. Thus, patients with myocarditis after BNT162b2 vaccination displayed a hallmark of decreased IFN (type I and type III) signaling and increased IL-6 production, which resembles the characteristics of bronchial epithelial cells. Peripheral immunity facilitates the landscape of patients with severe SARS-coV-2 infection^6,9^. Recently, the use of recombinant type I IFN as a treatment for COVID-19 was reported in a clinical trial^8,35^. After booster vaccination in healthy adults, IFNγ secretion increases in CD8 and CD4 T cells^36^. Meta-analysis in COVID-19 patients with or without severe symptoms also reported that patients with severe COVID-19 have a high IL-6/IFNγ ratio^37^. Therefore, IFN signaling in patients with myocarditis after vaccination appears to be similar to that in patients with severe COVID-19.

Viral myocarditis is a combination of direct cardiomyocyte damage and immune-mediated cell death^38^. In particular, NK cells play a crucial role in defense against acute viral pathogens, such as coxsackievirus B and murine cytomegalovirus^39^. In patients with severe COVID-19, the activity of NK cells in peripheral blood and bronchoalveolar lavage (BAL) increased, although the IFN response was blunted because the NK population was redistributed to BAL as a result of increased chemokines in BAL in patients with severe COVID-19^40,41^. High chemokine levels are also present in the plasma of patients with myocarditis by COVID-19, and NK cells can potentially be attracted to cardiomyocytes^42^. However, the number of NK cells in COVID-19 patients with severe symptoms is negatively correlated with IFNγ concentration^41^. In this study, we verified decreased IFN signaling in the NK cells of patients with severe side effects of BNT162b2 vaccination by analyzing epigenomic profiles. In future studies, more attention should be paid to IFN signaling in NK cells in myocarditis.

## Methods

### Ethics statement

The study was conducted following the Declaration of Helsinki and approved by the Institutional Review Board of Uijeongbu St. Mary’s Hospital (UC19TIDE0142). Written informed consent was obtained from all participants.

### Sample preparation

Blood was collected in ethylenediaminetetraacetic acid-coated tubes and mixed with the same amount of phosphate-buffered saline (PBS). Blood with PBS was then transferred to a leucosep tube. After centrifugation at 1,000 × g for 15 min at room temperature, the supernatant originating from the blood was collected in a 50 ml conical tube. The cells from the supernatant were washed twice by centrifugation at 400 × g for 10 min at room temperature. The supernatant was removed. Cells were counted and resuspended in a solution (1:9 DMSO: Fetal bovine serum). After 24 h in a cell container in a –80 °C deep freezer, the stock was stored in a liquid nitrogen tank.

### scATAC-seq (macrogen)

LUNA-FL Automated Fluorescence Cell Counter (Logos Biosystems) was used to consult the 10 × Genomics Single Cell Protocols Cell Preparation Guide and the Guidelines for Optimal Sample Preparation Flowchart (Documents CG00053 and CG000126, respectively) for more information on the cell preparation. The prepared cells were used for nuclei isolation according to the guidelines (Documents CG000169). Nuclei suspensions were incubated in a Transposition Mix that included transposase. Libraries were prepared using a chromium controller according to the 10 × Chromium Single Cell ATAC protocol (CG000209). Transposed nuclei were mixed with master mix and loaded with single-cell ATAC gel beads and partitioning oil into a chromium chip H. Transposed DNA fragments from single cells were uniquely barcoded within each droplet during thermal incubation. The barcoded DNA fragments were pooled in one tube and subjected to sample-index PCR. The purified libraries were quantified using qPCR according to the qPCR Quantification Protocol. Guide (KAPA) and qualified using an Agilent Technologies 4200 TapeStation (Agilent Technologies). The libraries were then sequenced using the HiSeq platform (Illumina) according to the read length in the user guide.

### scRNA-seq processing

The scRNA-seq dataset, SRR18209602 and SRR18209603, was obtained from a previous study. Datasets were counted with the cellranger v6.1^43^ pipeline using a human reference dataset (GRCh38) of 10X Genomics^43^. Subsequently, datasets were preprocessed with Seurat v4.2.0^44^ to remove low-quality cells using the following options: 200 < nFeatures < 4000, nCount < 15,000, percent.mt < 5. The filtered counts were normalized using the SCTransform^45^ function, with regression of the mitochondrial and ribosomal gene percentages. We used the FindIntegrationAnchors and IntegrateData commands for canonical correlation analysis (CCA) of Seurat to correct the batch effect. After integration, counts were log-normalized with the NoramlizeData function in Seurat and scaled with the ScaleData function with the default setting. The RunUMAP function was used with the first 30 PCs identified in the elbow plot to analyze the dimensional reduction. Clustering was performed using FindNeighbors and FindClusters functions, with a resolution of 0.6. Differential expression between cell types and samples was assessed with the Seurat FindMarkers function for genes detected in at least 10% of cells, higher than 0.25 logFC and lower than 0.05 FDR. The cluster annotation was performed in two steps. Annotation was performed by mapping to the azimuth human PBMC reference dataset using the FindVariableFeatures and FindTransferAnchors functions of Seurat. The initial annotation was corrected according to marker gene expression. Because the scRNA-seq expression matrix is ​​sparse, the imputation of missing values was performed for visualization using the expression recovery algorithm ALRA^46^.

### scATAC-seq processing

scATAC-seq datasets were counted with the cellranger-atac 2.1.0^47^ pipeline using a human reference dataset (GRCh38) from 10X Genomics. Subsequently, datasets were preprocessed with Signac v1.8.0^48^ to remove low-quality cells with the following options: 3000 < peak region fragments < 30,000, 15 < pct reads in peaks, 2 < TSS enrichment, nucleosome signal < 4. The filtered counts were normalized using the RunTFIDF function, in which frequency-inverse document frequency (TF-IDF) normalizes across cells to correct for differences in cellular sequencing depth and across peaks to give higher values to more rare peaks. Before integration, we created a common peak set across datasets using the reduce function of the GenomicRanges package and quantified peaks in each dataset using the Signac FeatureMatrix. Dimensional analysis was performed using singular value decomposition of the TF-IDF matrix after merging the data. To correct the batch effect among datasets, the Harmony v0.1.0^49^ package was used with 2–50 latent semantic indexing (LSI). The first LSI component was confirmed to be a technical variation through the DeptCor of Signac. To analyze dimensional reduction, the RunUMAP function was used with 2–20 LSIs identified in the elbow plot. Clustering was performed using FindNeighbors and FindClusters functions, with a resolution of 0.6. To quantify the accessibility of chromatin associated with each gene, a gene activity matrix was produced by counting the number of fragments intersecting the gene body and promoter region using protein-coding genes annotated in the Ensembl database (EnsDb.Hsapiens.v86). Gene activity was log-normalized and scaled before annotation. Differential chromatin accessibility between cell types and samples was assessed with the Signac FindMarkers function for gene activities detected in at least 10% of cells and higher than 0.25 logFC and lower than 0.05 FDR. The gene closest to each of the differentially accessible peaks was defined using the ClosestFeature function of Signac. Cluster annotation was performed in two steps. Label transfer was conducted using an existing scRNA-seq dataset as a reference using the FindTransferAnchors and TransferData functions of Seurat. The initial annotation was corrected according to the marker gene activity and lineage-specific motif activity. Genomic regions containing scATAC-seq peaks were annotated, except for clusters without significant DAR, with ChIPSeeker 1.32.1^50^ and clusterProfiler 4.4.4^51^ using the UCSC database on hg38.

### Motif analysis (chromVAR)

We performed Motif analysis using choromVAR v 3.3.2^19^. Motif information was added to the peak matrix by Signac AddMotifs using motif position frequency matrices from the JASPAR 2020 database. ChromVAR activities were calculated using the RunChromVAR wrapper in Signac after matching the set of background peaks. The differential activity was computed using the FindMarker function. We performed TF footprinting using the Footprint of Signac^48^ and visualized the PlotFootprint to predict the binding location of a TF.

### Cis-element co-accessibility

We constructed cis-co-accessible networks (CCANs) with Cicero v 1.3.0^33^ from the scATAC-seq peak. The Seurat object was converted to the CellDataSet (CDS) format of Monocle3 using the as.cell_data_set function of the SeuratWrappers package. CCAN, calculated using the run_cicero function of monole3, utilized a k-nearest-neighbors approach, which creates overlapping sets of cells.

## Supplementary Information

Supplementary Information.

## Data Availability

scATAC seq data and single-cell VDJ analysis relevant to the manuscript have been uploaded to the Sequence Read Archive (SRA) BioProjectID PRJNA910983 (Reviewer link: https://dataview.ncbi.nlm.nih.gov/object/PRJNA910983?reviewer=banqjkfk0slvf5oojvl5vro65g).
