# Supplementary material for: Epigenomic landscape exhibits interferon signaling suppression in the patient of myocarditis after BNT162b2 vaccination
Source: Sci Rep. 2023 Jun 1;13:8926. doi: 10.1038/s41598-023-36070-y (PMC10234245; doi:10.1038/s41598-023-36070-y)
Supplement: Supplementary file 1 — Supplementary Information. [file 41598_2023_36070_MOESM1_ESM.docx]

**Supplemental file**

**
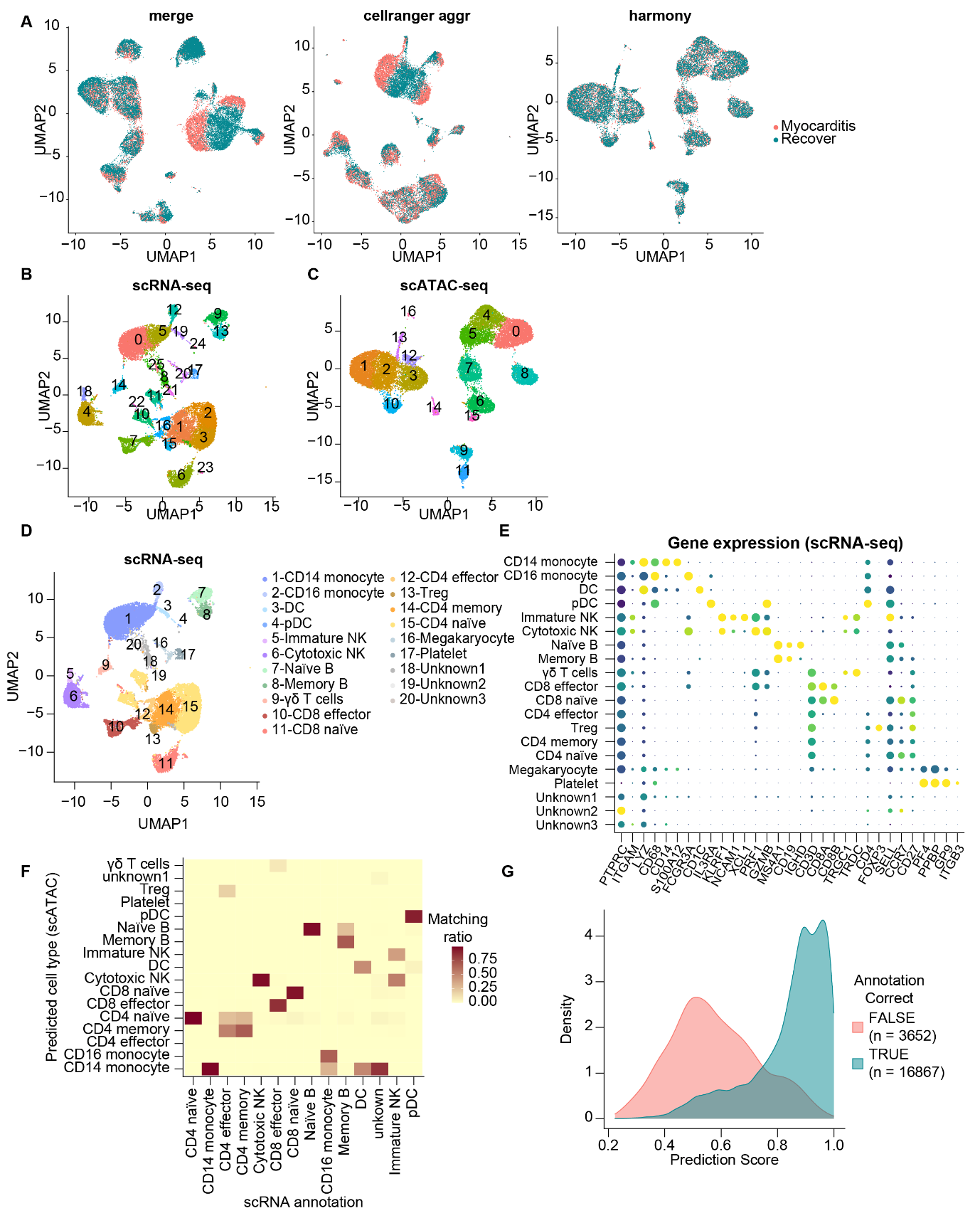
**

Supplementary Figure 1.

(A) Uniform manifold approximation and projection (UMAP) plot of single-cell sequencing assay for transposase-accessible chromatin (scATAC-seq) according to different integration methods. Each sample was presented as a different color.

(B) Uniform manifold approximation and projection (UMAP) UMAP plot representing the scRNA-seq Seurat clusters.

(C) Uniform manifold approximation and projection (UMAP) plot representing sea clusters of single-cell sequencing assay for transposase-accessible chromatin (scATAC-seq).

(D) Uniform manifold approximation and projection (UMAP) plot representing cluster annotation of scRNA-seq.

(E) Dot plot showing canonical immune cell marker gene expression in scRNA-seq datasets. The diameter corresponds to the population percentage of cells expressing genes in the subtype. The average gene expression level of the cell subtype appears as a color gradation.

(F) Heatmap representing the matching ratio of each cluster population between annotated scRNA-seq clusters and label-transferred cluster annotations of single-cell sequencing assay for transposase-accessible chromatin (scATAC-seq).

(G) The concordance ratio represents the true or false ratio between the scRNA-seq annotation and predicted label-transferred annotation of single-cell sequencing assay for transposase-accessible chromatin (scATAC-seq) using label transfer from the scRNA-seq annotation.


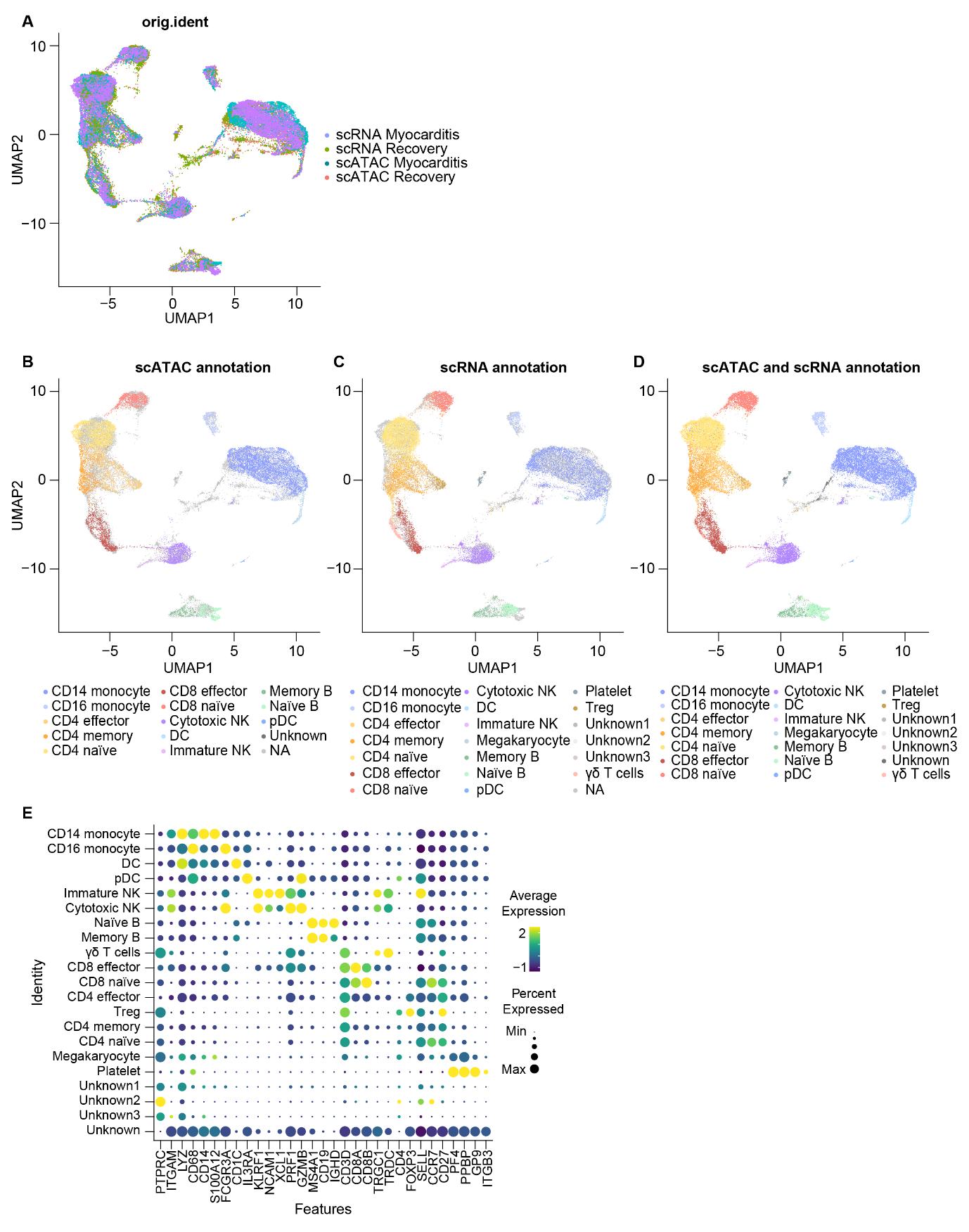


Supplementary Figure 2.

(A) Uniform manifold approximation and projection (UMAP) plot representing co-embedding of scRNA-seq and single-cell sequencing assay for transposase-accessible chromatin (scATAC-seq) datasets in a single plot.

(B, C, D) Co-embedding uniform manifold approximation and projection (UMAP) overlaid with the annotation of scATAC, scRNA, and scATAC/scRNA, respectively.

(E) Dot plot showing canonical immune cell marker gene activity and imputed RNA expression in the coding dataset. The diameter corresponds to the population percentage of cells expressing genes in the subtype. The integrated average gene expression level of the cell subtype appears as a color gradation.


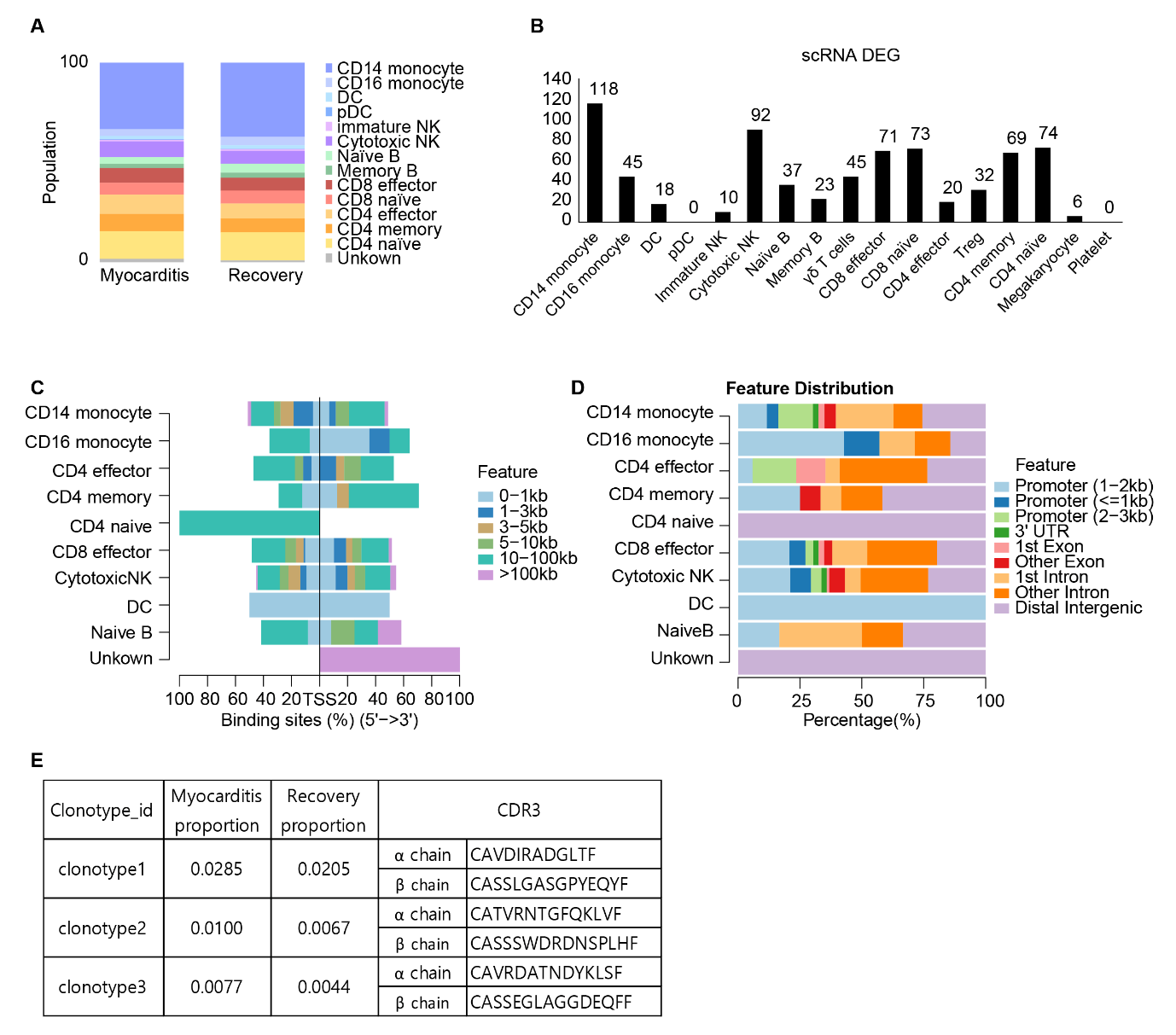


Supplementary Figure 3.

(A) Cluster fraction by sample state was calculated using 10x-based single-cell sequencing assay for transposase-accessible chromatin (scATAC-seq).

(B) The number of differentially expressed genes (DEGs) in myocarditis versus recovery in each immune cell subtype was counted (logFC > 0.25, adjusted P value < 0.05, minimum percentage of expressing cells > 10% ).

(C) The Distance from differentially accessible region (DAR) to the transcription start site (TSS) of the nearest gene was calculated for each immune cell type.

(D) DAR location was annotated for each immune cell type.

(E) Top clone-type TCR across conditions.


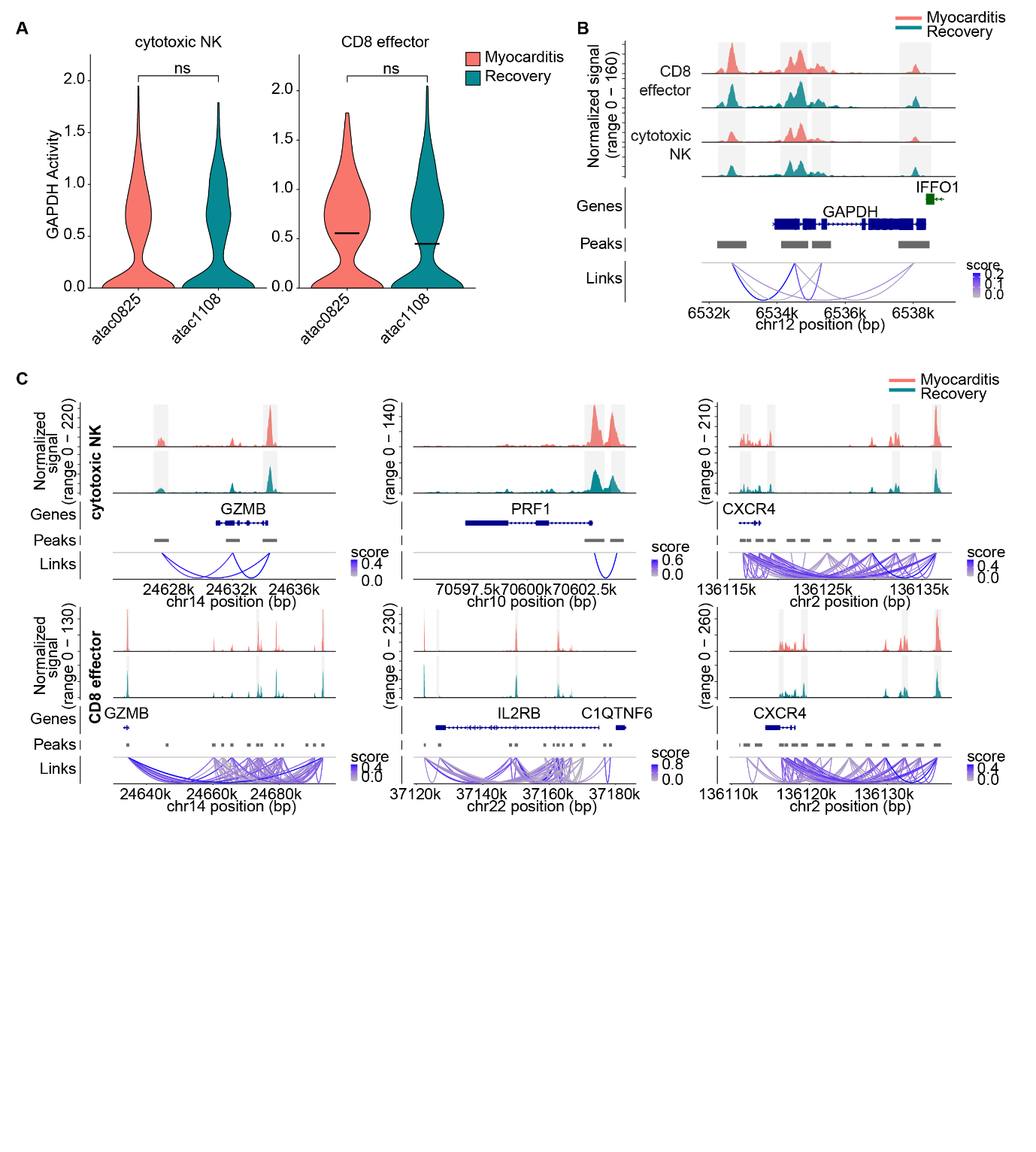


Supplementary Figure 4.

(A) Violin plot showing the GAPDH gene activity in the cytotoxic NK and CD8 effector clusters.

(B) Aggregated genome tracks of GAPDH locus (top) with the CCAN score (bottom).

(C) Aggregated genome tracks of immune response activation marker gene locus and upstream (top) with the CCAN score (bottom) in the cytotoxic NK and CD8 effector clusters.


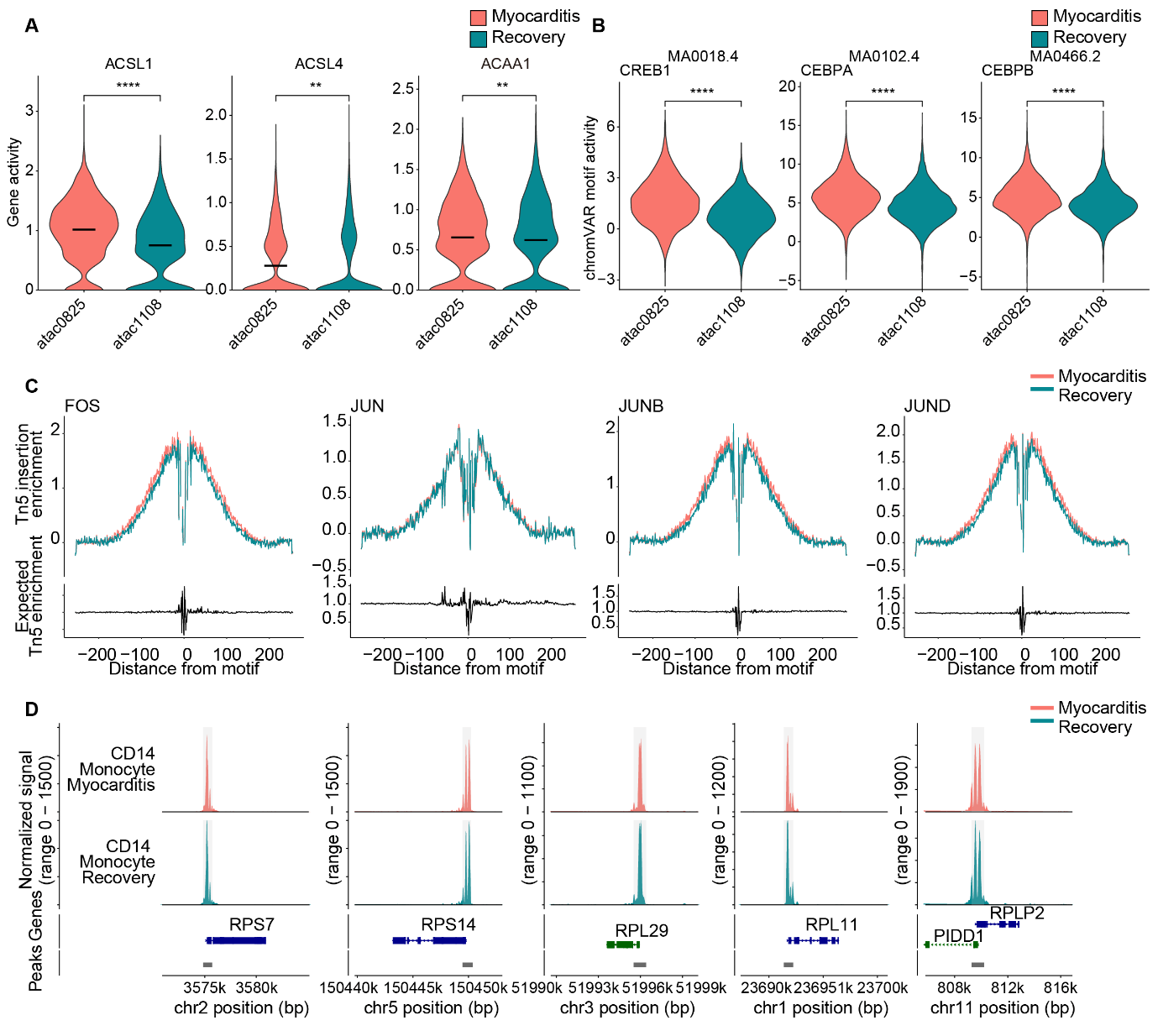


Supplementary Figure 5.

(A) Violin plot showing the fatty acid metabolism related gene activity in the CD14 monocyte.

(B) Violin plot showing the CREB1, CEBPA, and CEBPB chromVAR activity in the CD14 monocyte.

(C) TF footprints of FOS, JUN, JUNB, and JUND in the CD14 monocyte subtypes. The Tn5 insertion bias track is also shown.

(D) Aggregated genome tracks of JUN/FOS target gene locus.


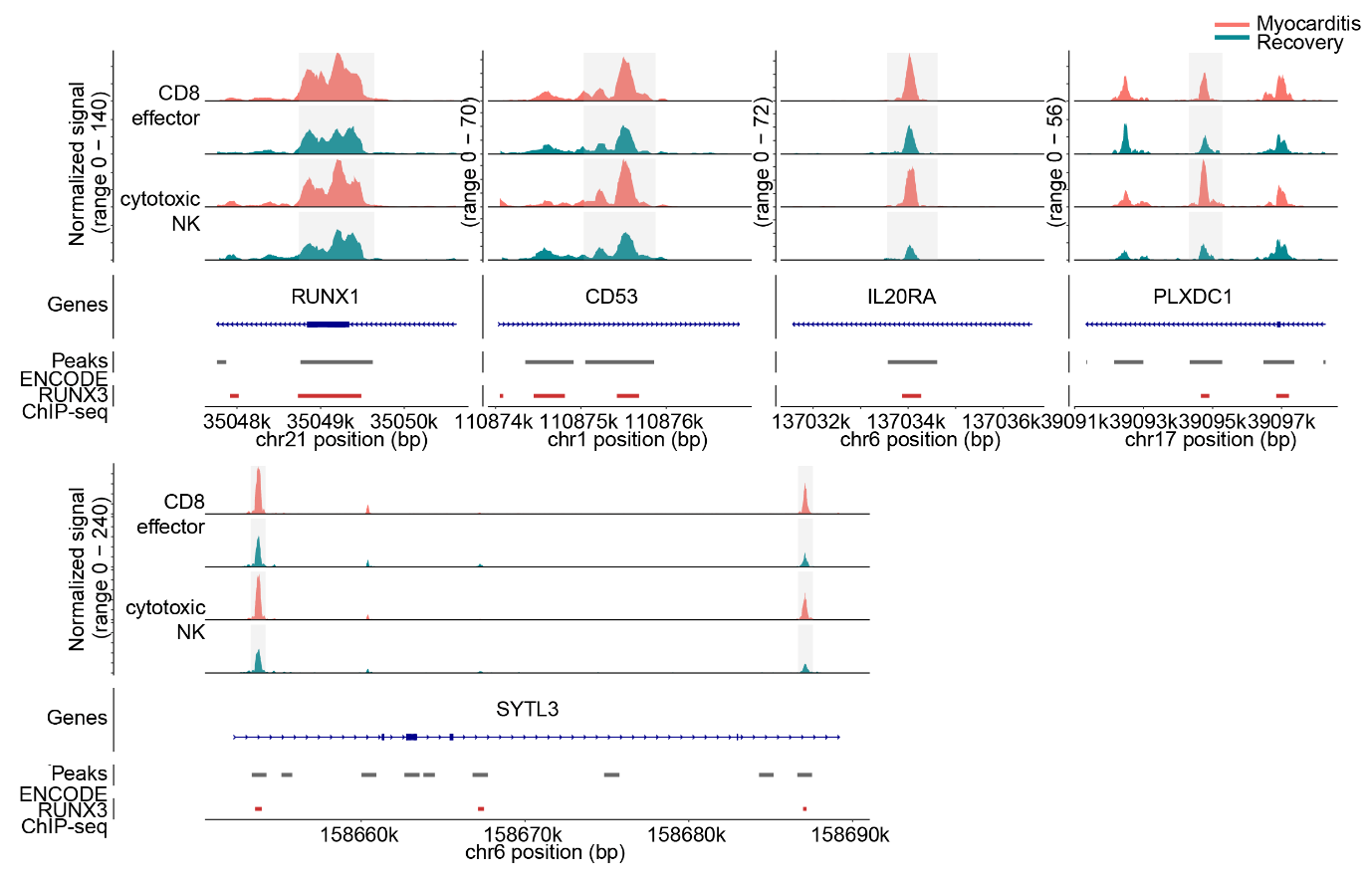


Supplementary Figure 6. Aggregated genome tracks of each gene locus (top) with peak positions (middle) and ENCOD RUNX3 ChIP-seq peak positions (bottom).


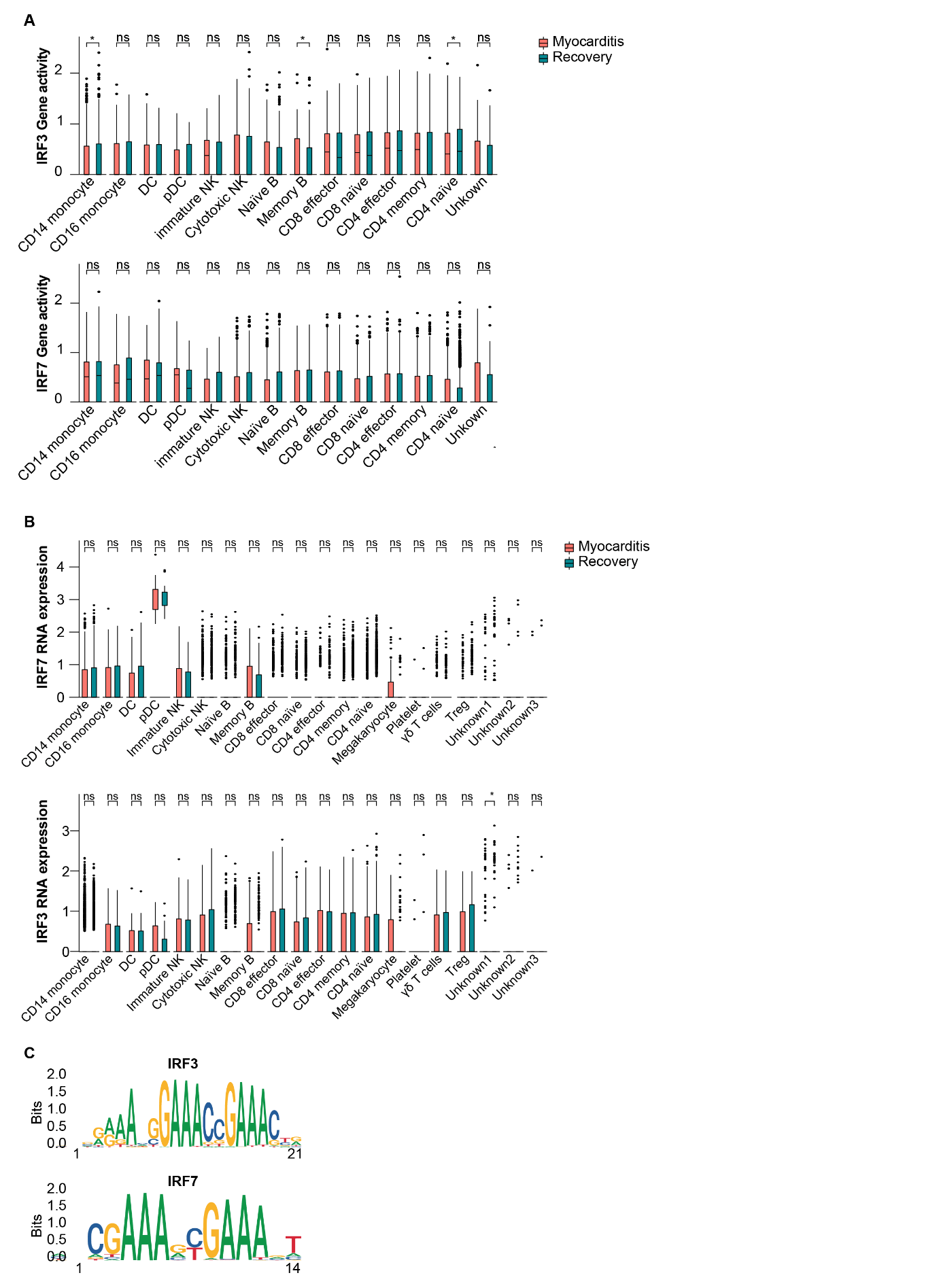


Supplementary Figure 7

(A) Box plot showing IRF3 and IRF7 gene activity in the immune cell subtype.

(B) Box plot showing IRF3 and IRF7 RNA expression in the immune cell subtype.

(C) Plot of the position weight matrices for the IRF3 and IRF7 motifs.


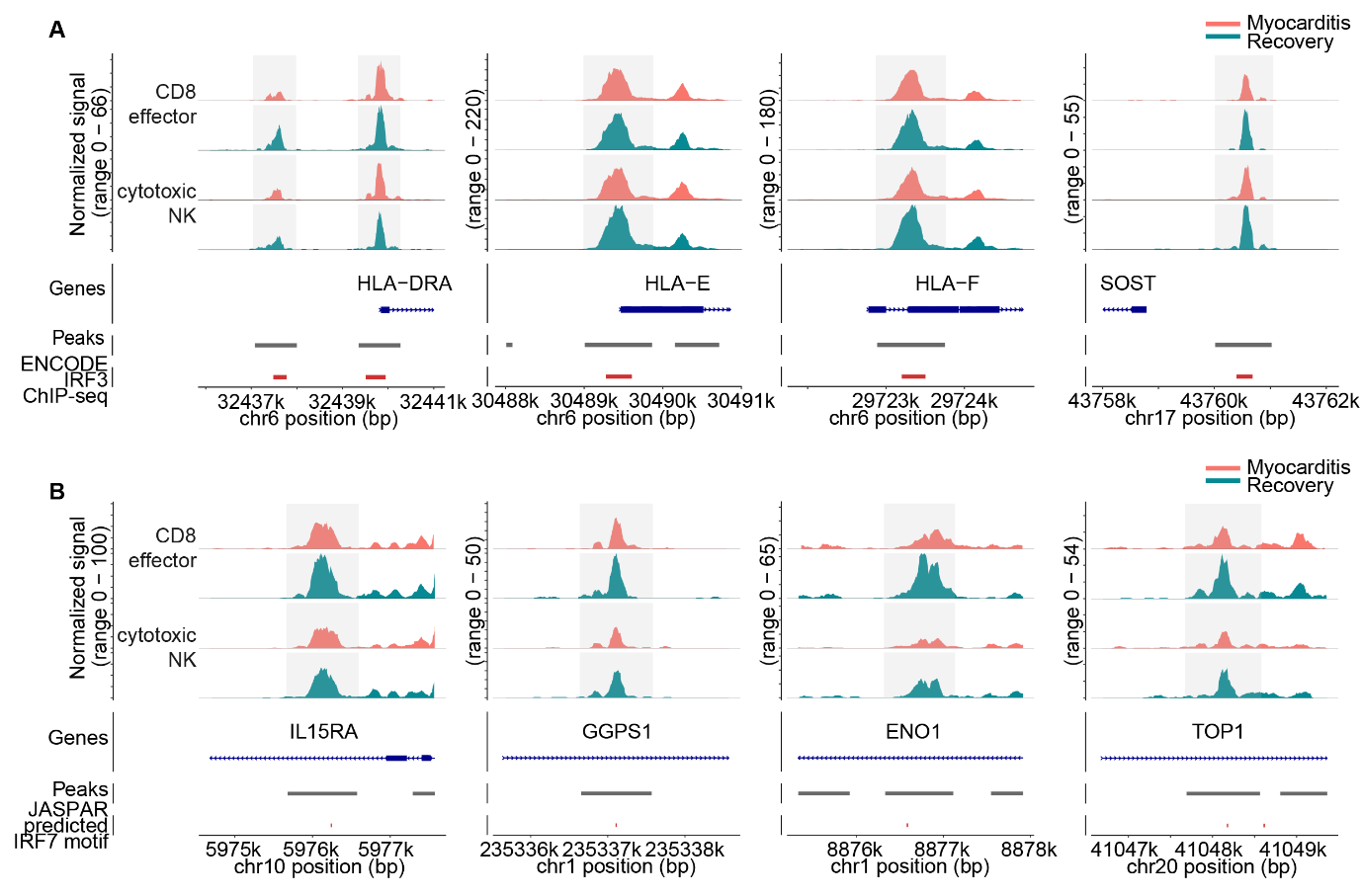


Supplementary Figure 8.

(A) Aggregated genome tracks of each gene locus (top) with peak positions (middle) and ENCOD IRF3 ChIP-seq peak positions (bottom).

(B) Aggregated genome tracks of each gene locus (top) with peak positions (middle) and IRF7 motif locations predicted by JASPAR score (bottom).


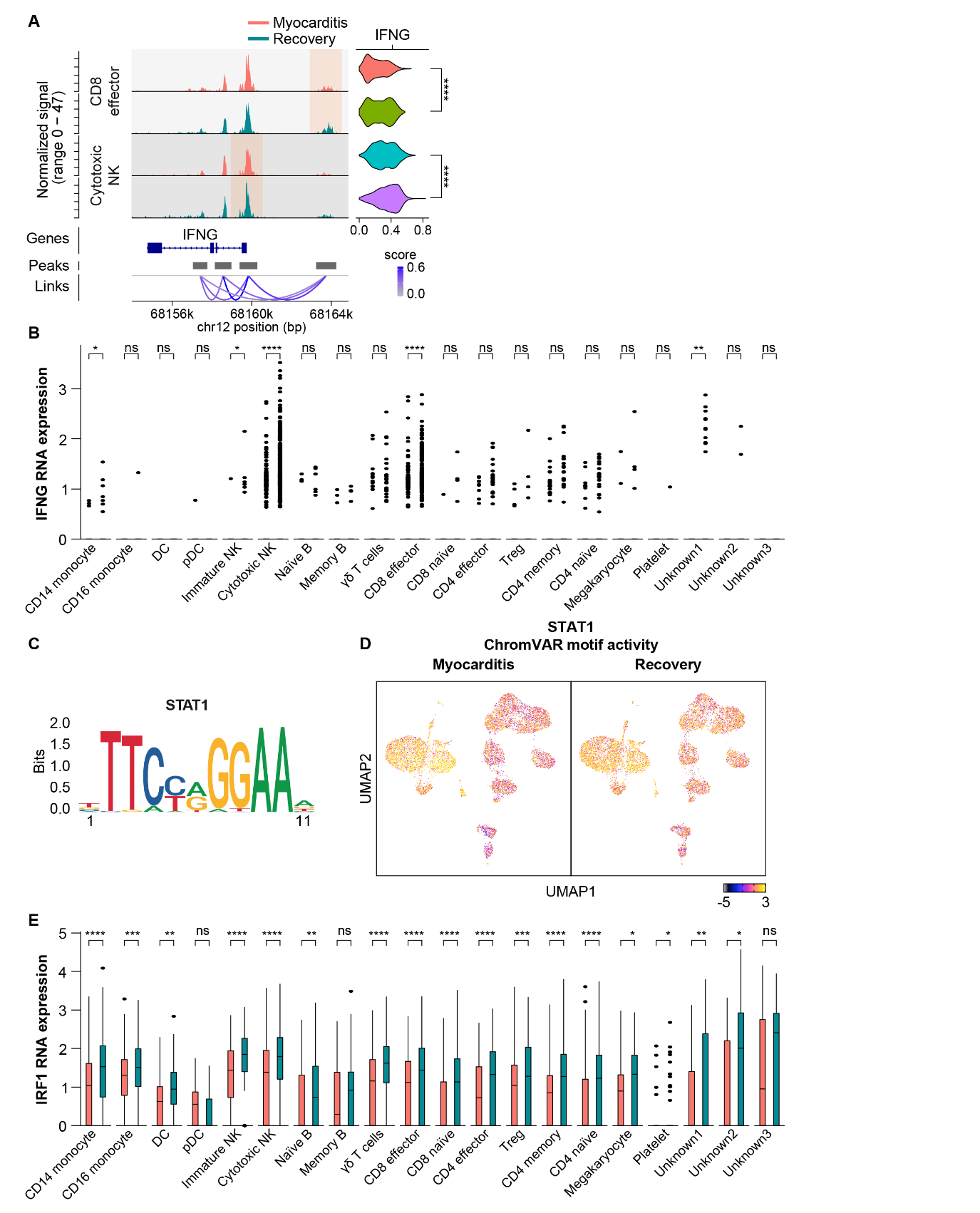


Supplementary Figure 9.

(A) Aggregated genome tracks of the IFNG loci and upstream region (top, left) with CCAN score (bottom) and violin plot of IFNG RNA expression (top, right).

(B) Box plot showing IFNG gene expression for the immune cell subtype.

(C) Plot of the position weight matrices for the motifs of STAT1.

(D) The uniform manifold approximation and projection (UMAP) plot of single-csell sequencing assay for transposase-accessible chromatin (scATAC-seq) overlaid with the motif activity of STAT1. The color gradient represents the chromVAR TF motif bias-corrected deviations.

(E) Box plot showing IRF1 gene expression in the immune cell subtype.


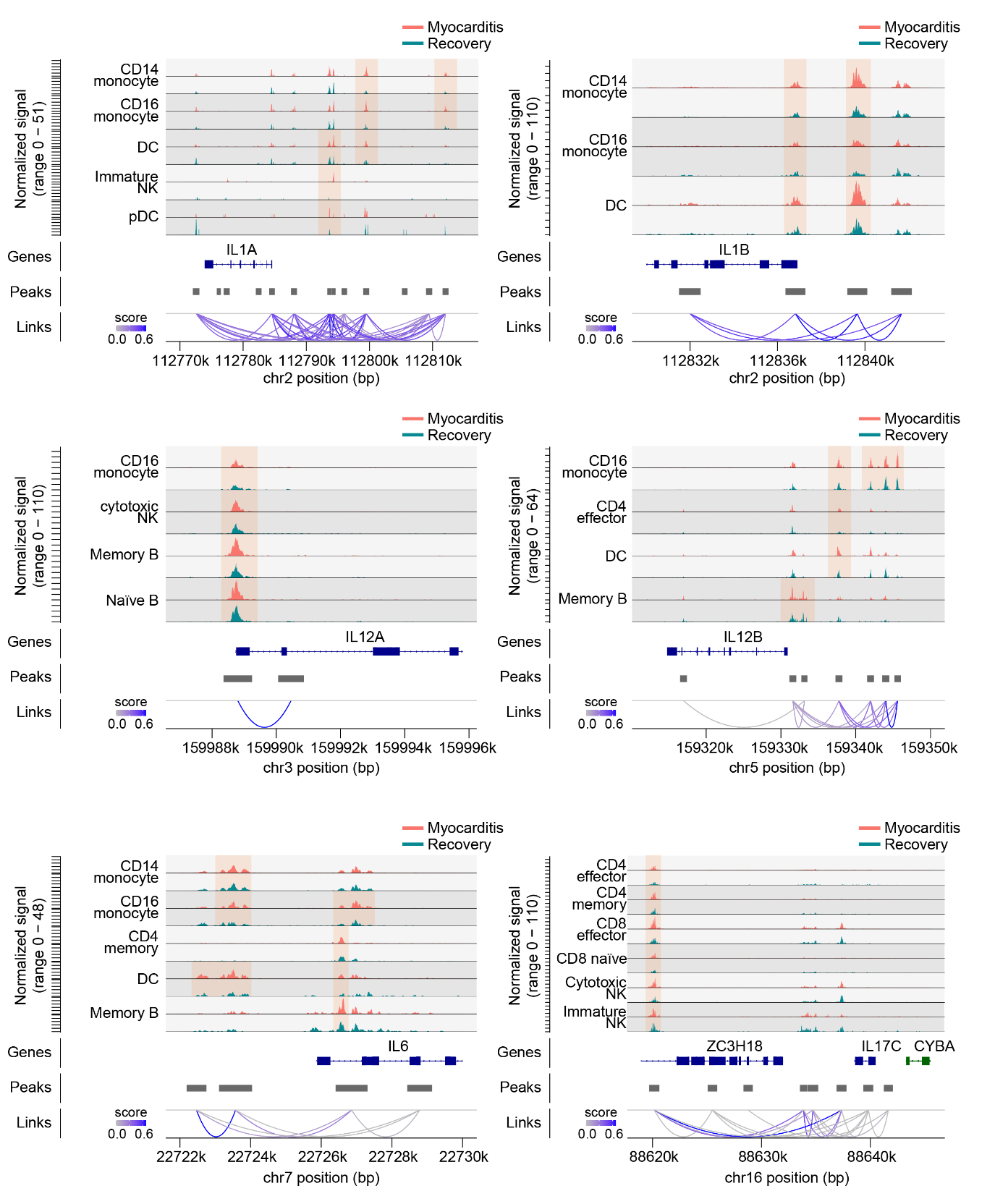


Supplementary Figure 10. Aggregated genome tracks of each gene locus and upstream (top) with the CCAN score (bottom).
